# The Hospital World

**Published:** 1911-01-28

**Authors:** 


### The Hospital World.

New and old supporters of the Wimbledon Hospital in Kingston Road will be glad to hear that in the rebuilding scheme—which, by the way, the King's Fund has generously supported—the name of a past worker is to be honoured in the same manner as that of the late King. Two wards, in short, are to be dedicated, one to his late Majesty, the other to the memory of Dr. Poole Collyns, who was the first medical officer to the institution. This memorial of him comes perhaps with the greater meaning from the fact that his death a few years ago has not lessened the respect which his work created among his colleagues.

